# Endoscopic Retrieval of Multiple Elongated Swallowed Foreign Bodies in a Patient With Psychiatric Illness

**DOI:** 10.7759/cureus.96027

**Published:** 2025-11-03

**Authors:** Jahnavi Pasila, Charlotte Sumner, Kehkashan Anwar, Mamoon Solkar, Nafees Qureshi

**Affiliations:** 1 General Surgery, Tameside General Hospital, Manchester, GBR; 2 Surgery, Tameside General Hospital, Manchester, GBR

**Keywords:** case report, endoscopic retrieval, foreign body ingestion, intentional ingestion, non-surgical removal, oesophagogastroduodenoscopy (ogd), psychiatric illness, toothbrush ingestion

## Abstract

Foreign body ingestion is common, with most passing uneventfully through the gastrointestinal tract. However, ingested long and rigid foreign bodies pose a significant challenge for endoscopic removal from the oesophagus and stomach. This case report describes a 19-year-old woman with a history of psychiatric illness who swallowed 12 elongated foreign objects, which were successfully retrieved via esophago-gastro-duodenoscopy (EGD). To our knowledge, this is one of the first documented case reports of multiple swallowed foreign bodies managed endoscopically without the need for surgical intervention.

## Introduction

Accidental ingestion of foreign bodies in adults can often be linked to underlying factors, such as alcoholism, bulimia nervosa, rapid eating without adequate mastication and mental illness. The diagnosis typically relies on a thorough history, clinical assessment, and radiological examination, with various foreign bodies identifiable on abdominal radiographs in emergency departments [1]. While most ingested small foreign bodies pass naturally through the gastrointestinal tract and are eventually excreted in the feces, long and sharp objects present unique challenges due to their shape.

Toothbrushes, pencils, and mascara wands are rare foreign bodies that can be accidentally or intentionally ingested. Their unusual shapes make spontaneous passage through the gastrointestinal tract theoretically impossible. Consequently, there are very few documented cases of these specific objects passing naturally. When foreign bodies cannot pass, complications such as pressure necrosis, perforation, bleeding, and gastrointestinal ulceration may arise, potentially leading to life-threatening sepsis [2].

Given these risks, early removal of ingested foreign bodies if not passable through the gastrointestinal tract is crucial to prevent complications. Here, we present a rare case involving multiple elongated foreign objects, specifically toothbrushes, broken pencils, and mascara wands, which were successfully managed through endoscopy.

## Case presentation

This is the case of a 19-year-old female who was brought to the Emergency Department at our hospital from a psychiatric ward by her nurses, after ingesting a toothbrush intentionally several days prior and a broken pencil a few hours before arrival. The patient had a history of autism and psychosis, as well as a previous history of battery ingestion that required endoscopic removal. Upon presentation, four hours post-ingestion, she reported epigastric discomfort and foreign body sensation in her throat. She also had pain during swallowing (odynophagia), which had been bothering her for the previous couple of days. Her hemodynamic status was stable with a normal pulse, blood pressure and oxygen saturation. Her National Early Warning Score (NEWS) at presentation was 0. 

**Figure 1:**
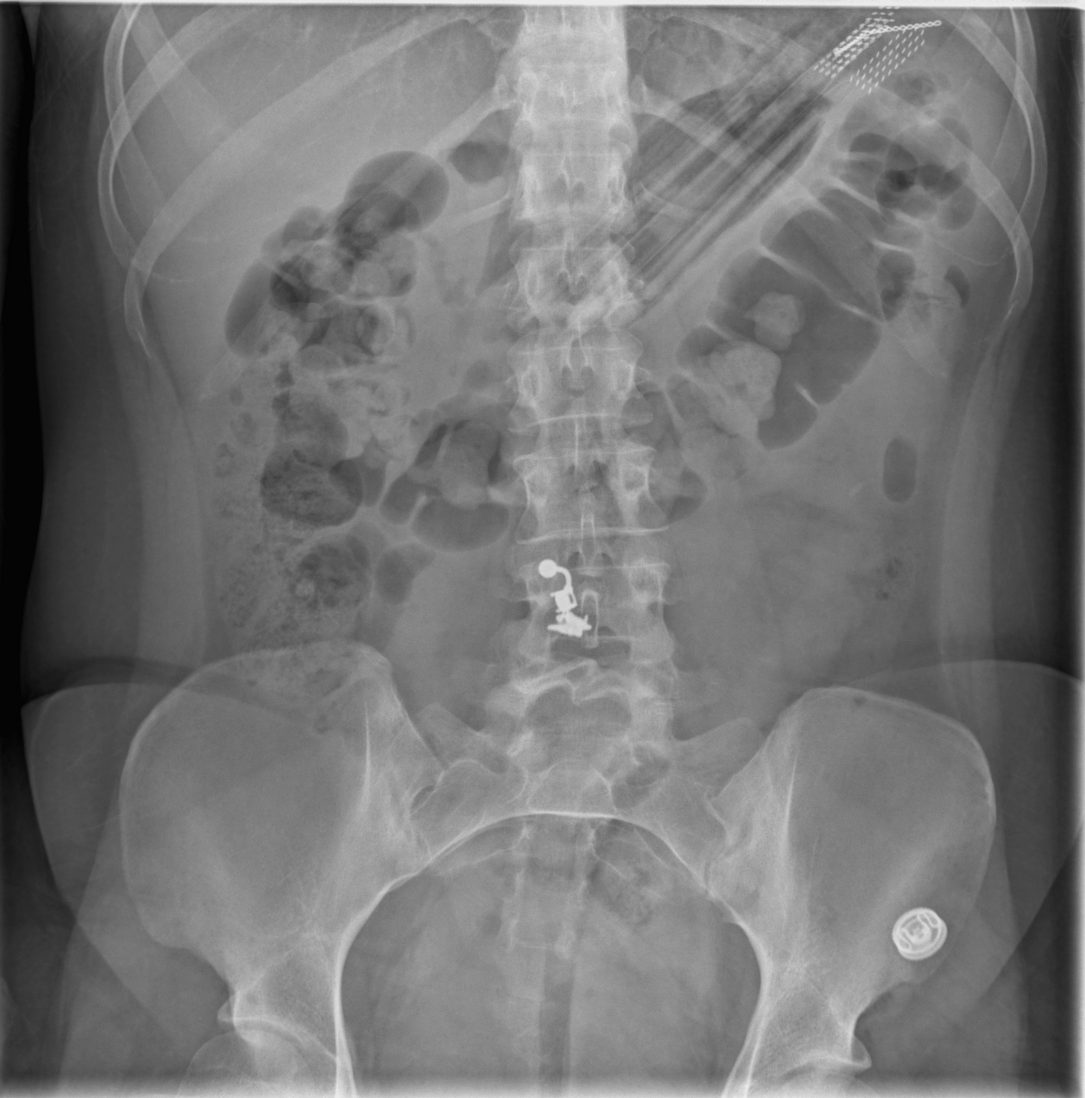
Abdominal X-ray confirming the presence of two toothbrushes in the left upper quadrant identified by their bristles.

General physical examination revealed a soft abdomen with tenderness localised to the epigastric region on deep palpation, with no signs of peritonism. An urgent abdominal X-ray confirmed the presence of the toothbrush in the upper left quadrant as shown in Figure 1. She was admitted under the surgical team for observation and was reviewed by the admitting consultant surgeon the following morning, who recommended endoscopic retrieval of the foreign body. Due to her cognitive limitations, the patient was provided with information appropriate to her level of comprehension, and a consent form (Form 4) was signed on her behalf by her mother for an esophago-gastro-duodenoscopy (EGD) for foreign body retrieval, with the possibility of surgical intervention if endoscopic removal proved unsuccessful. Informed consent for publication of this case report and accompanying images was obtained from the patient’s legal guardian.

An initial EGD was performed under general anaesthesia with an endotracheal tube in situ (4mg midazolam) two days after admission, with retroflexion of the scope in the stomach and successful visualisation of the upper gastrointestinal tract up to the second part of the duodenum (D2), which appeared normal. The ingested toothbrush and pencil were visualised. However, removal of these objects using a snare was unsuccessful due to the perpendicular attachment of the toothbrush, causing the snare wire to become entangled with the foreign body, necessitating it to be cut during the procedure and left in the stomach for further procedure under general anaesthesia as the patient became distressed. Given the inability to remove the foreign body under conscious sedation, a second EGD was scheduled. 

**Figure 2:**
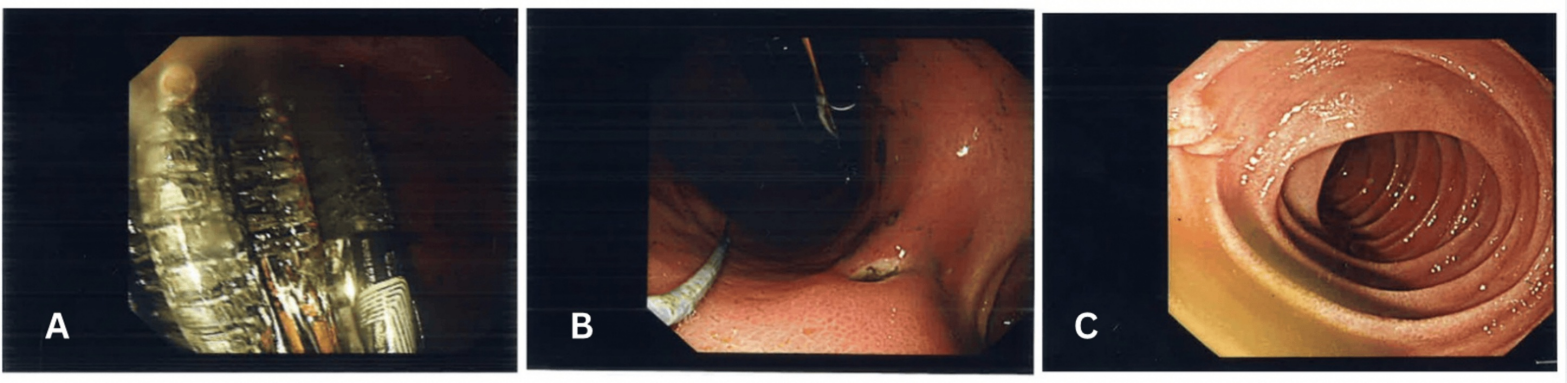
Endoscopic findings during retrieval of ingested foreign bodies (A) Insertion of the snare with visualisation of three toothbrushes and part of a makeup pencil head in the stomach. (B) Presence of an antral ulcer at the level of the antrum, likely secondary to mucosal trauma from the foreign bodies. (C) Visualisation of the first part of the duodenum (D1) with evident plica circularis and no mucosal abnormalities.

**Figure 3:**
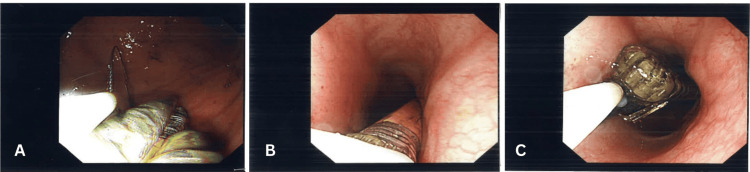
Endoscopic extraction sequence of ingested foreign bodies. (A) Visualisation of the snare loop engaged around one of the makeup brushes during retrieval. (B) Retrieval of the foreign body from the gastroesophageal junction. (C) Successful extraction of the makeup brush and pencil through the oesophagus under direct endoscopic visualisation.

**Figure 4:**
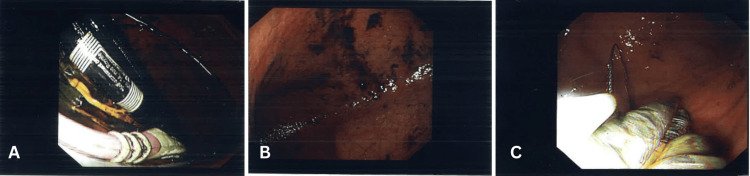
Extraction of foreign bodies at the level of the gastroesophageal junction (GEJ). (A) Multiple elongated foreign bodies, including a mascara tube and toothbrush being maneuvered toward the gastroesophageal junction. (B) Endoscopic view of mucosal contact and minor surface erythema at the GEJ following manipulation. (C) Controlled extraction of the foreign body through the GEJ using a snare under direct endoscopic guidance.

**Figure 5:**
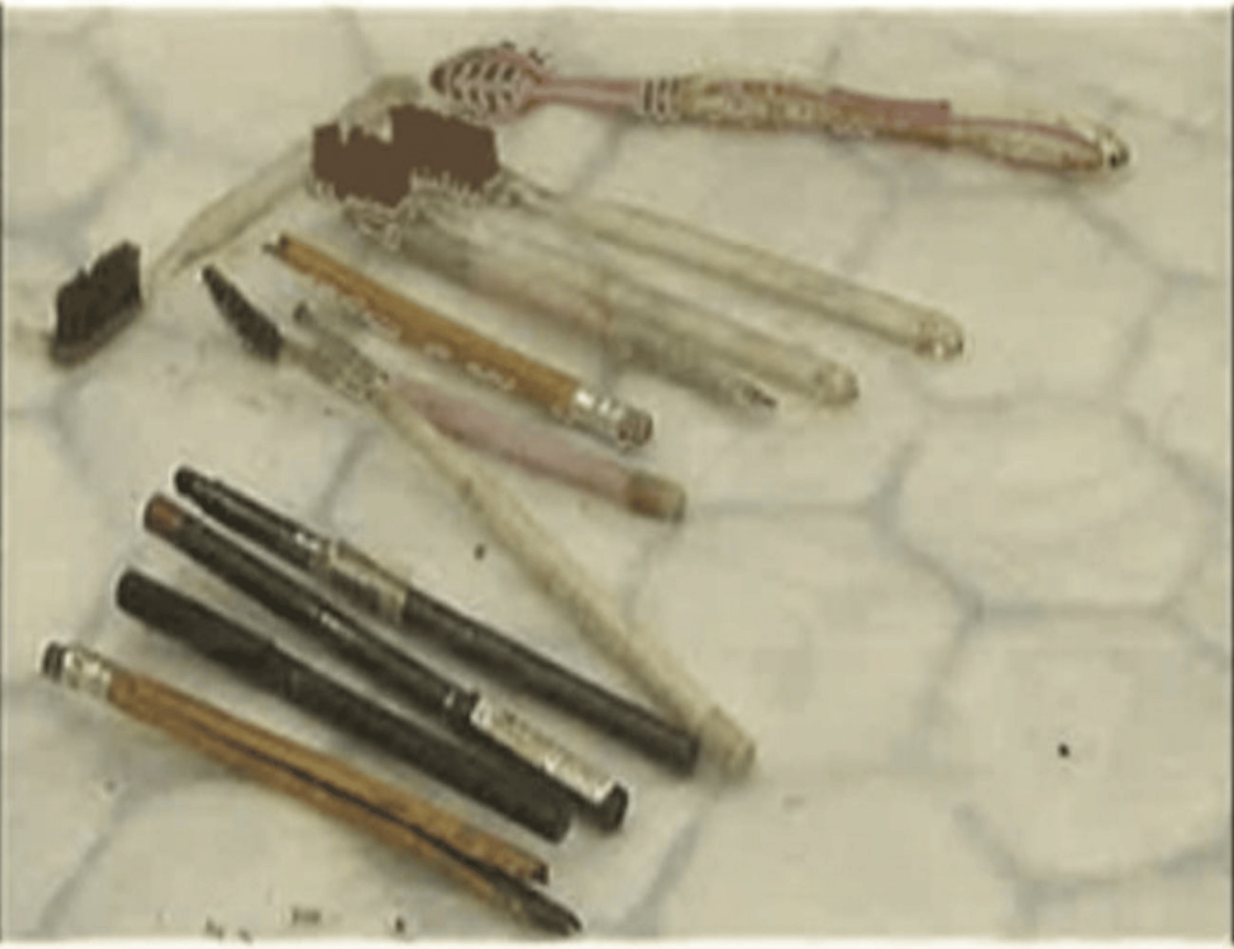
Image displaying all 12 extracted foreign bodies, including four long toothbrushes, two broken lead pencils, and six makeup brushes/mascara tubes.

The repeat procedure was performed under general anaesthesia two days later. The patient was positioned supine, and an EGD scope (size 28) was advanced up to D2. While the oesophagus appeared normal, an antral ulcer (Figure 2) had developed likely secondary to the presence of the multiple foreign bodies found in the stomach, including the leftover snare wire from the recent EGD (Figure 3). Retrieval of the foreign bodies was achieved using a snare and grasping forceps, chosen according to the size and position of each object. The entire procedure lasted approximately 45 minutes to an hour. Extraction was pictured at the level of gastroesophageal junction (GEJ) (Figure 4) during which four long toothbrushes (approximately 19cm), two broken lead pencils (each approximately 8.5x8.5cm), and six makeup brushes/mascara tubes (approximately 12cm) were also removed (Figure 5).

All 12 foreign objects and the leftover snare wire were successfully retrieved with multiple passes and without immediate or early complications or signs of perforation. Each object was retrieved with a single pass. 

The patient had an uneventful recovery following the successful endoscopic retrieval of 12 elongated foreign bodies and no intra-procedural complications occurred. Post-procedural monitoring revealed no adverse events and was discharged back to the psychiatric unit two days post-procedure. A follow-up EGD was arranged for six to eight weeks to assess the healing of the identified antral ulcer. She re-presented a few weeks later with a recurrence of intentional ingestion, this time involving another toothbrush. A repeat EGD was performed, and the object was removed without complication. The patient was subsequently discharged with continued psychiatric follow-up and risk management planning to prevent further episodes. 

## Discussion

Patients who ingest foreign bodies may present with or without symptoms. When symptomatic, they may experience odynophagia, dysphagia, nausea, vomiting, and chest or abdominal pain [3]. Sharp objects can lead to bleeding or perforation, while large objects may cause gastric outlet or bowel obstruction. 

While most ingested foreign bodies pass spontaneously, endoscopic intervention is needed in approximately 20% of cases, and surgery is required in less than 1% [4]. However, compared to accidental ingestion, in cases of intentional ingestion, the rate of endoscopic intervention is notably higher, ranging from 63-76% [5]. Research indicates that most cases of foreign-body ingestion are intentional, often occurring in patients with psychiatric conditions who engage in this behaviour repeatedly [6]. 

Common anatomical sites where foreign bodies may become lodged include the cricopharyngeal area, gastroesophageal junction, pylorus, and ileocecal valve. The location and size of the object impact both the treatment approach and the method of removal. Initial management includes assessing the patient’s respiratory status and identifying any signs of respiratory compromise [5]. Radiologic and endoscopic evaluations are often necessary, with plain X-rays serving as a valuable early tool to help not only localise the object but also classify the number and size. In some cases, further detailed imaging with CT is required. These images may indicate complications such as perforation [4]. 

Typically, management follows a conservative approach, progressing to endoscopy and finally surgery if needed. Endoscopy serves as a minimally invasive bridge to surgery when conservative management fails, offering the benefits of cost savings and reduced hospital stay. Delays between ingestion, presentation, and intervention can increase the risk of requiring surgery and experiencing complications [7].

There have been many recorded case reports of toothbrush/pencil ingestion [1,2]; however we have not seen a single case report where 12 or more elongated objects have been retrieved endoscopically. Few studies have reported ingestion of longer objects or ingestion of multiple objects involving shorter and sharper objects [8]. In this case, the patient ingested 12 foreign bodies, such as toothbrushes, pencils, or mascaras, resulting in persistent abdominal pain, odynophagia, and nausea. She had a history of foreign body ingestion and similar hospital admissions. 

It seems advisable to categorize ingested bodies by material, size and surface consistency, as these characteristics help determine urgency of intervention. Foreign bodies longer than 6cm and wider than 2.5cm are particularly difficult to pass beyond the duodenum. Urgent endoscopy is recommended for objects greater than 6cm in length, ideally within 12 to 24 hours, to reduce the risk of aspiration, esophageal obstruction, impaction, and perforation [4]. 

The patient was initially referred to the surgical team after reporting the ingestion of a toothbrush and a pencil. However, during endoscopy, additional foreign objects were discovered. It was subsequently revealed that the patient had been engaging in this behavior over an extended period, without disclosing these incidents to the psychiatric nursing staff. 

In this case, the initial endoscopic retrieval attempt was unsuccessful when the endoscopic snare became entangled with the foreign object and had to be detached and left in situ. The patient's family was consulted about further treatment options, including a repeat endoscopic attempt versus surgical intervention. Ultimately, a decision was made to attempt endoscopy again, this time by a surgeon, with operative treatment onboard if necessary. 

The second endoscopic attempt succeeded in removing each object as well as the broken snare with individual passings without causing trauma or on-table complications. Based on this case, we recommend that clinicians consider using flexible endoscopic retrieval performed by an experienced endoscopist. While an overtube was not used in this case, its use may be beneficial in similar situations to protect the oesophageal mucosa, facilitate multiple insertions, and reduce the risk of aspiration or mucosal injury during retrieval of multiple or elongated foreign bodies. When multiple foreign bodies are present, the procedure should preferably be performed under general anaesthesia to ensure airway protection, patient safety, and optimal procedural control. If a gastroenterologist performs the procedure under general anaesthesia in the theatre, an on-call surgeon should be available on-site in case surgery is needed. Alternatively, involving a surgeon in the initial procedure may be advisable when the risk of injury or perforation is high. Importantly, obtaining consent for potential surgical intervention prior to any endoscopic attempt is essential, allowing for prompt surgical exploration if endoscopy is unsuccessful and avoiding delays or patient distress [7]. 

## Conclusions

Intentional ingestion of foreign objects is often seen in psychiatric patients, with many instances still necessitating surgical intervention, unlike this particular case, where they were extracted endoscopically. Because of their irregular shape, foreign bodies that are long, rigid and sharp such as toothbrushes, cutlery, pens, etc. are particularly unlikely to pass spontaneously and often require endoscopic or surgical removal as they cannot pass through the gastrointestinal tract naturally, presenting a difficult clinical challenge, with only a few cases documented in the literature. Timely extraction is essential to avoid impaction in the duodenum and lessen the risk of complications. Overall, it is important to highlight that endoscopic removal in these situations should be conducted by a skilled endoscopist to ensure safe extraction. 

## References

[1] Soga M, Tanaka T, Ueda T (2022). Accidental duodenal foreign body of toothbrush removed laparoscopically: a case report. Surg Case Rep.

[2] Al-Mulla AE, Buhamad F, Marafie HF, Al Khalifa F (2023). A successful endoscopic extraction of accidently ingested toothbrush in an adult: a case report. J Surg Case Rep.

[3] Qureshi N, Cherian N, Solkar M, Ben-Hamida A (2016). Endoscopic retrieval of an intentionally ingested mobile phone in an adult: first case report of its kind. Ann Clin Case Rep.

[4] Ambe P, Weber SA, Schauer M, Knoefel WT (2012). Swallowed foreign bodies in adults. Dtsch Arztebl Int.

[5] Ikenberry SO, Jue TL, Anderson MA (2011). Management of ingested foreign bodies and food impactions. Gastrointest Endosc.

[6] Palta R, Sahota A, Bemarki A, Salama P, Simpson N, Laine L (2009). Foreign-body ingestion: characteristics and outcomes in a lower socioeconomic population with predominantly intentional ingestion. Gastrointest Endosc.

[7] Obinwa O, Cooper D, O'Riordan JM (2016). An ingested mobile phone in the stomach may not be amenable to safe endoscopic removal using current therapeutic devices: a case report. Int J Surg Case Rep.

[8] Te Wildt BT, Tettenborn C, Schneider U, Ohlmeier MD, Zedler M, Zakhalev R, Krueger M (2010). Swallowing foreign bodies as an example of impulse control disorder in a patient with intellectual disabilities: a case report. Psychiatry (Edgmont).

